# 1413. Skin microbiome: The Profile of Organisms and the Effect of Skin Decolonization.

**DOI:** 10.1093/ofid/ofad500.1250

**Published:** 2023-11-27

**Authors:** Diana Fernández-Rodríguez, Jeongeun Cho, Emanuele Chisari, Javad Parvizi

**Affiliations:** MD/PhD Plan de Estudios Combinados en Medicina (PECEM), Mexico City, Distrito Federal, Mexico; Rothman Orthopaedic Institute, Philadelphia, Pennsylvania; Rothman Orthopaedic Institute, Philadelphia, Pennsylvania; Rothman Orthopaedic Institute, Philadelphia, Pennsylvania

## Abstract

**Background:**

The most common organisms causing surgical site infection (SSI) are frequently found as part of the skin microbiota. Decolonization of the skin prior to a surgical procedure has been shown to be effective in reduction of SSI. The aim of this prospective study was to determine the organism profile of the skin and evaluate the effect of the application of an antiseptic solution on the skin microbiome.

**Methods:**

A total of 50 volunteers were recruited into this study. After randomization, one arm was treated with a pre-saturated wipe containing a benzalkonium chloride (BZK)-based antiseptic solution and the contralateral arm was wiped with a PBS-based pre-saturated wipe. Swab samples of each extremity were taken at baseline (prior to application of the agents) and at 4 different timepoints after application (5 min, 2 h, 24 h, and 1 month). Skin was protected between 5 min and 2 h after application with a sterile wrap to prevent environmental contamination between sampling. Microbiological analysis consisted of standard culture and Next-generation sequencing.

Study design

**Figure:**
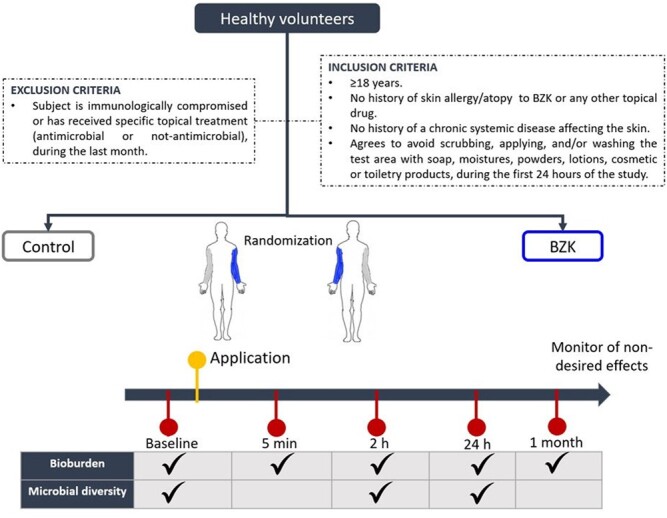

Fifty volunteers were recruited for this double-blinded clinical trial. Randomization was performed to determine which arm will take the benzalkonium chloride (BZK) testing solution. We assessed bioburden and microbial diversity at 4 different timepoints after application (5 minutes, 2 hours, 24 hours, and 1 month).

**Results:**

The baseline skin bioburden varied greatly among individuals (393 [IQR, 87-2 016] CFU/ml); however, baseline bioburden within subjects did not differ between the arms (p=0.61). A higher effect of bioburden reduction was observed at 5 minutes and 2 hours after application of BZK (log_10_ 1.314 ± 0.086 and log_10_ 1.308 ± 0.104, respectively; p< 0.01), compared to PBS. By 24 hours, the reduction in bacterial load was also higher in the BZK treated arm (log_10_ 0.445 ± 0.115; p< 0.01). The top species affected by the treatment were *C. acnes, S. cohnii, E. cloacae, L. crispatus*, among others. The relative abundance of all *Staphylococcus* species was significantly decreased after the application of BZK, from 34.50% to 20.48%, at the 2-hour timepoint (p< 0.01).

Bacterial alpha diversity metrics between groups and timepoints assessed (baseline, 2 hours, and 24 hours).

**Figure:**
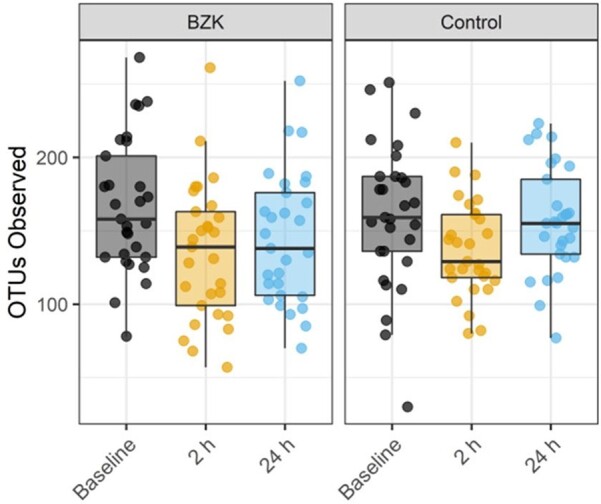

Bacterial composition.

**Figure:**
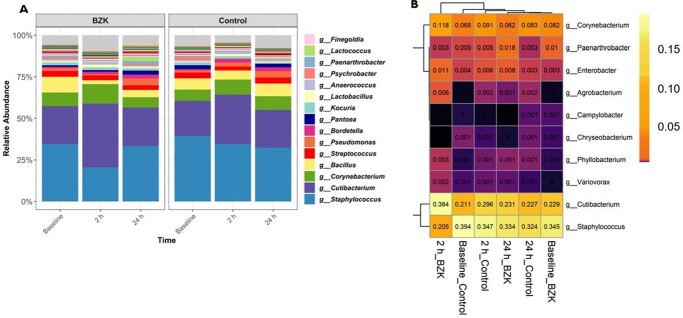

A) Relative abundance of the most prevalent bacterial genus and B) heatmap illustrating the mean relative abundance of bacteria detected to be differentially abundant by ANCOMBC procedure. A cell colored in black can be considered “true” zero, whereas there were no detections of a particular bacteria for that given cell.

CFU log10 reduction, from baseline bioburden, at different timepoints after application of the testing solutions (BZK or PBS). Data is expressed as mean ± SEM.

**Figure:**
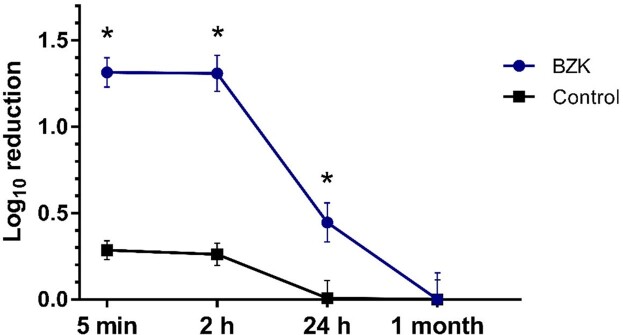

**Conclusion:**

There was a wide difference in the skin microbiome between individuals but the organism profile was very similar in two arms of a given individual. Application of the BZK-based antiseptic solution led to a substantial reduction of the skin flora for up to 24 hours after application but the organism profile returned towards baseline rapidly. It appears that antiseptic solutions applied to skin are capable of transiently and markedly reduce the bioburden of skin

**Disclosures:**

**Javad Parvizi, MD, FRCS**, 3M: Grant/Research Support|Acumed, LLC: Stocks/Bonds|Aesculap: Grant/Research Support|Alphaeon: Stocks/Bonds|AO Spine: Stocks/Bonds|Becton Dickenson: Advisor/Consultant|Biomet: Grant/Research Support|Cardinal Health: Advisor/Consultant|Cempra: Grant/Research Support|CeramTec: Grant/Research Support|Ceribell: Stocks/Bonds|Coracoid: Stocks/Bonds|Corentec: Advisor/Consultant|Datatrace: Grant/Research Support|DePuy: Grant/Research Support|Elsevier: Grant/Research Support|Elute: Stocks/Bonds|Ethicon: Advisor/Consultant|Hip Innovation Technology: Stocks/Bonds|Illuminus: Stocks/Bonds|Integra: Grant/Research Support|Intellijoint: Stocks/Bonds|Jaypee Publishers: Grant/Research Support|KCI / 3M (Acelity): Advisor/Consultant|Lima: Grant/Research Support|MicroGenDx: Advisor/Consultant|Molecular Surface Technologies: Stocks/Bonds|Myoscience: Grant/Research Support|Nanooxygenic: Stocks/Bonds|National Institutes of Health (NIAMS & NICHD): Grant/Research Support|NDRI: Grant/Research Support|Novartis: Grant/Research Support|OREF: Grant/Research Support|Orthospace: Grant/Research Support|Osteal: Stocks/Bonds|Parvizi Surgical Innovations and Subsidiaries: Stocks/Bonds|Peptilogic: Stocks/Bonds|Peptilogics: Advisor/Consultant|Pfizer: Grant/Research Support|PRN-Veterinary: Grant/Research Support|Rotation Medical: Grant/Research Support|Simplify Medical: Grant/Research Support|SLACK Incorporated: Grant/Research Support|Smith & Nephew: Grant/Research Support|Sonata: Stocks/Bonds|Stelkast: Grant/Research Support|Stryker: Grant/Research Support|Synthes: Grant/Research Support|Tenor: Advisor/Consultant|TissueGene: Grant/Research Support|Tornier: Grant/Research Support|Wolters Kluwer Health - Lippincott Williams & Wilkins: Grant/Research Support|Zimmer Biomet: Advisor/Consultant|Zimmer Biomet: Grant/Research Support

